# A Systematic Review of Pain Catastrophizing and Chronic Musculoskeletal Pain

**DOI:** 10.1016/j.pmn.2025.07.014

**Published:** 2025-08-25

**Authors:** Sotaro Shimada, Ardith Z. Doorenbos, Ellen Goldstein, Dahee Wi

**Affiliations:** College of Nursing, University of Illinois Chicago, Chicago, IL

**Keywords:** Catastrophizing, Chronic pain, Musculoskeletal pain, Pain measurement, Pain intensity, Pain interference

## Abstract

**Objectives:**

This study was designed to synthesize recent randomized controlled trials examining the associations between pain catastrophizing and four key pain-related outcomes (i.e., pain intensity, disability, pain interference, and physical function) among adults with chronic musculoskeletal pain. This review clarifies the role of pain catastrophizing in pain outcomes to inform targeted interventions.

**Design:**

A systematic review was performed following the Preferred Reporting Items for Systematic Reviews and Meta-Analyses guidelines.

**Methods:**

The Cumulative Index of Nursing and Allied Health Literature, PubMed, Excerpta Medica Database, PsycINFO, Scopus, and Web of Science were searched. The key search terms “pain catastrophizing,” “chronic musculoskeletal pain,” and “pain-related outcomes” were combined to find randomized controlled trials published in English from October 2018 to 2024 and study quality was assessed using the revised Cochrane Risk-of-Bias tool for randomized trials. The data were descriptively synthesized.

**Results:**

After screening, 20 studies were included in the review. The major pain type was chronic back pain. Among the studies, 13 reported a significant association between pain catastrophizing and pain-related outcomes, with some studies identifying multiple significant outcomes, including pain intensity (n = 8), disability (n = 7), and pain interference (n = 3).

**Conclusion:**

This review highlights the associations between pain catastrophizing and pain-related outcomes in adults with chronic musculoskeletal pain. The findings emphasize the importance of addressing pain catastrophizing in interventions to improve pain-related outcomes.

**Funding:**

This work was supported by Grant K24 AT011995 from the National Center for Complementary and Integrative Health and the National Institute of Neurological Disorders and Stroke.

Chronic musculoskeletal pain, most commonly low back and neck pain, is the primary cause of disability worldwide (Ferrari et al., 2024). It accounts for a large proportion of chronic pain overall (Rometsch et al., 2024). Chronic pain has been related to various unfavorable health outcomes such as anxiety, depressive disorder, activity limitation, and decreased quality of life (Aaron et al., 2025; Cáceres-Matos et al., 2020; Hadi et al., 2019). Furthermore, healthcare treatment related to chronic pain and the loss of worker productivity due to pain have tremendously impacted the United States (Centers for Disease Control and Prevention, 2025). Consequently, chronic musculoskeletal pain impacts both individual lives and society as a whole.

Previous studies have reported that an individual’s psychological characteristics, such as anxiety and depression, are associated with pain-related outcomes, including pain intensity, pain interference, disability, and physical function (Gerdle et al., 2023; Silveira et al., 2024). In particular, pain catastrophizing is considered to be a critical psychological factor associated with pain-related outcomes (Martinez-Calderon et al., 2021; Rogers & Farris, 2022). Pain catastrophizing is defined as the repetitive development of exaggerated and negative cognitive and emotional schemas characterized by rumination, magnification, and helplessness in response to actual or anticipated pain (Flink et al., 2013; Quartana et al., 2009). Pain catastrophizing has been found to act as a mediator between pain intensity and depression among adults with chronic noncancer pain (Liu et al., 2024); between race and/or ethnicity and pain-related outcomes, such as pain intensity and disability in adults with knee osteoarthritis (Fullwood et al., 2021); and between pain intensity and pain interference among patients enrolled in an interdisciplinary pain rehabilitation program (Schumann et al., 2022).

A better understanding of the associations between pain catastrophizing and pain-related outcomes can facilitate a greater understanding of chronic musculoskeletal pain, which in turn can contribute to better clinical decision-making for the treatment of patients with chronic musculoskeletal pain. Two previous review studies have identified associations between pain catastrophizing and both pain-related outcomes and psychological conditions (Martinez-Calderon, Jensen, et al., 2019; Rogers & Farris, 2022). However, one review focused on comprehensive chronic pain, and the other review, although focused on musculoskeletal pain, was published 5 years ago and did not include randomized clinical trials (RCTs).

The previous systematic review (Martinez-Calderon, Jensen, et al., 2019) showed significant positive associations between pain catastrophizing and both pain intensity and disability in cross-sectional analyses, as demonstrated through meta-analyses. Although meta-analyses were not conducted for longitudinal associations due to heterogeneity of included studies, the evidence suggested consistent associations between pain catastrophizing at baseline and subsequent pain intensity and disability.

Building upon these findings, the current review focuses on expanding the evidence by incorporating recent longitudinal studies and examining the potential mediating and moderating roles of pain catastrophizing in intervention outcomes for adults with chronic musculoskeletal pain. The present study exclusively focused on RCTs to include relatively high-quality articles. The RCT is one of the most rigorous study designs for minimizing potential biases (Hariton & Locascio, 2018) and to satisfy a temporally ordered structure and experimental manipulation, which are rarely met in observational studies.

The present review was designed to provide a more comprehensive understanding of the longitudinal relationships between pain catastrophizing and pain-related outcomes by integrating the findings with the previous systematic review (Martinez-Calderon, Jensen, et al., 2019), and the synthesis may suggest that pain catastrophizing is associated with pain-related outcomes in addition to influencing treatment responsiveness and long-term prognosis, which ultimately inform the development of more targeted and effective pain management strategies. The present systematic review, focused on RCTs published within the past 5 years, aims to examine the evidence of associations between pain catastrophizing and four pain-related outcomes—pain intensity, disability, pain interference, and physical function—among adults with chronic musculoskeletal pain. The review was conducted to clarify the role of pain catastrophizing as a potential predictor, moderator, or mediator, thereby providing insights into for whom and how interventions may be most effective based on the most current evidence.

## Methods

### Design

This is a systematic review following the Preferred Reporting Items for Systematic reviews and Meta-Analyses (PRISMA) guidelines (Page et al., 2021). The present review focuses exclusively on RCTs to critically synthesize evidence on the associations between pain catastrophizing and pain-related outcomes.

### Data Sources and Search Strategies

The data sources for the review included the Cumulative Index of Nursing and Allied Health Literature (CINAHL) Plus with Full Text, MEDLINE accessed via PubMed, Excerpta Medica Database (EMBASE) via Elsevier, APA PsycINFO via ProQuest, Scopus via Elsevier, and Web of Science via Clarivate. The search strategy was established based on previous reviews (Martinez-Calderon, Flores-Cortes, et al., 2019; Martinez-Calderon, Jensen, et al., 2019; Schütze et al., 2018) and the guidance of a health science research librarian (R.R.). Key search terms related to chronic musculoskeletal pain, pain catastrophizing, pain intensity, disability, pain interference, and physical function were identified. Since the most recent published review covered articles published through September 2018 (Martinez-Calderon, Jensen, et al., 2019), the current search was limited to articles published between October 1, 2018, and October 1, 2024, to capture the most recent and methodologically rigorous evidence. Given that the focus was exclusively on RCTs, the authors aimed to reflect current clinical practices and emerging research trends in pain management. The detailed search strategy for each database is provided in Supplementary File 1.

### Eligibility Criteria

Musculoskeletal pain was defined according to the Analgesic, Anesthetic, and Addiction Clinical Trial Translations, Innovations, Opportunities, and Networks and American Pain Society (ACTTION-APS) Pain Taxonomy for chronic pain (Dworkin et al., 2016), which includes diagnosed fibromyalgia, chronic myofascial pain, widespread pain, gout, osteoarthritis, rheumatoid arthritis, spondyloarthropathies, and spine pain. The review included RCTs (e.g., pragmatic controlled trials, pilot studies, and secondary analyses of RCTs) that included adults 18 years or older who had been diagnosed with chronic musculoskeletal pain, defined as persistent or episodic pain lasting more than 3 months. Eligible studies measured pain catastrophizing and pain intensity, disability, pain interference, and/or physical function and reported at least one statistical analysis of the association between pain catastrophizing and one of the selected pain-related outcomes. Furthermore, eligible RCTs had to assess pain catastrophizing with a reliable and validated measure (Simic et al., 2024), such as the Coping Strategies Questionnaire (CSQ)-Catastrophizing subscale (Rosenstiel & Keefe, 1983), the Pain Catastrophizing Scale (PCS) (Sullivan & Bishop, 1995), abbreviated versions of the PCS (McWilliams et al., 2015), or a translated version of the CSQ or PCS. Finally, eligible RCTs had to be published as peer-reviewed original articles in English with full text readily available. Studies that measured outcomes pre- and/or postsurgery were excluded, as surgical procedures are known to dynamically alter pain catastrophizing (Giordano et al., 2020). Consequently, such populations were not considered representative of the population of interest.

### Data Management and Selection

Covidence software (Veritas Health Innovation, Melbourne, Australia) was used to screen and manage the information extracted from the databases. After discarding duplicate studies, screening of titles and abstracts was performed to eliminate ineligible studies. Subsequently, S.S. and D.W. reviewed full-text articles to identify eligible studies. Discrepancies were discussed and resolved with the assistance of a third reviewer (A.D.).

### Data Extraction and Synthesis

S.S. abstracted the data from eligible studies. D.W. double-checked this process, and discrepancies were resolved by a third reviewer to achieve a consensus. The abstracted data included author, year, design, aim, population (e.g., age, pain type, pain duration, sample size), intervention and/or control, measurement of pain-related outcomes, association between pain catastrophizing and pain outcomes, limitations, and recommendations for future research. The principal findings were descriptively synthesized to demonstrate the associations between pain catastrophizing and pain-related outcomes, such as pain intensity, disability, pain interference, and physical function, among adults with chronic musculoskeletal pain. A meta-analysis was not conducted due to the heterogeneity of the study designs, sample characteristics, and interventions, which made it difficult to calculate an estimated effect size of pooled studies.

### Quality Assessment

For quality assessment, S.S. first assessed the risk of bias in the selected studies using the revised Cochrane Risk-of-Bias (RoB 2) tool for randomized trials (Sterne et al., 2019). This assessment was then checked and confirmed by D.W. and A.D. The Cochrane RoB 2 consists of five assessed domains: the randomization process, deviations from the intended interventions, missing outcome data, measurement of the outcome, and selection of the reported result. Each domain includes three to seven questions that can be answered with “Yes,” “Probably yes,” “Probably no,” “No,” or “No information.” Thereafter, judgment algorithms are applied to determine whether each domain presents “low risk,” “some concerns,” or “high risk.” Subsequently, the overall risk of bias in each study is identified by combining the assessment results for the five domains. In the case of the secondary analyses of RCT data included in this review, the authors were often required to locate another paper or protocol previously published or registered to obtain further methodological information related to the parent RCT.

## Results

### Search Results

**Figure 1.**
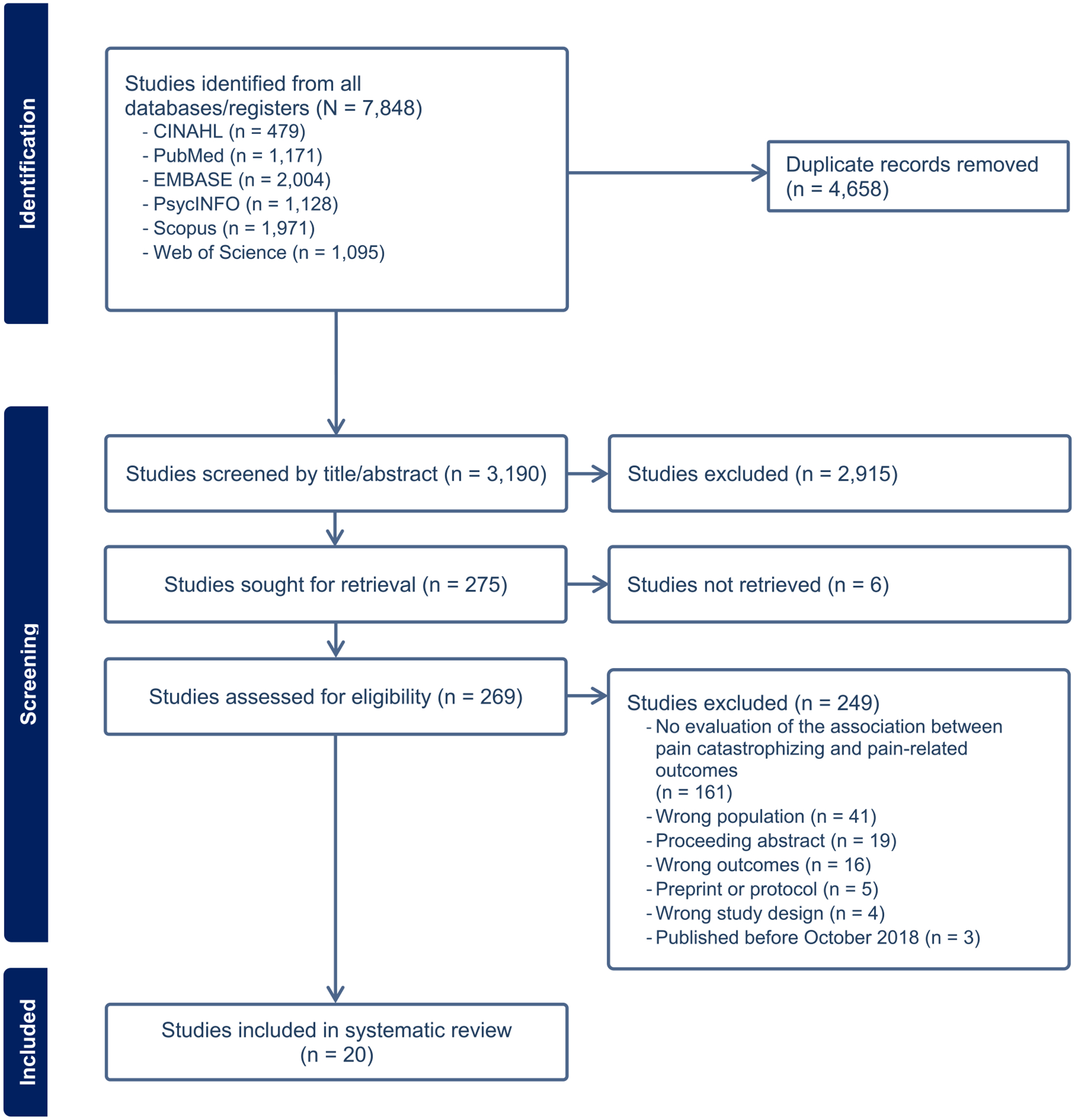
PRISMA flow diagram. CINAHL, Cumulative Index to Nursing and Allied Health Literature; EMBASE, Excerpta Medica Database.

A total of 7,848 articles were retrieved from the six electronic databases, and after excluding 4,858 duplicates, 3,190 articles underwent title and abstract screening. This process resulted in 269 full-text articles remaining for consideration. Among these, 249 were deemed ineligible for various reasons, such as a lack of examination of the association between pain catastrophizing and pain-related outcomes (n = 161) or a participant population that did not meet the eligibility criteria (n = 41). Consequently, 20 studies were selected for inclusion in this systematic review, as shown in Figure 1. In addition, nine parent RCT articles were manually obtained for further methodological information related to the included studies to assess the quality of studies.

### Study Characteristics

Table 1 summarizes the 20 studies included in this review. Ten studies reported RCTs, while the other ten studies reported secondary analyses of data from RCTs. The RCTs were conducted across eight countries: the United States (n = 6), Australia (n = 5), Belgium (n = 2), Brazil (n = 2), Iran (n = 2), Norway (n = 1), Spain (n = 1), and Sweden (n = 1). The study populations included adults with chronic back pain (including low back pain, n = 15), fibromyalgia (n = 2), chronic musculoskeletal pain (n = 1), chronic spinal pain (n = 1), and chronic neck pain (n = 1). The sample sizes ranged from 48 to 521, with a total of 3,316 participants across the included studies; the mean sample size was 165.8 (standard deviation = 134.5).

The 20 studies evaluated multiple interventions, including education-related interventions (n = 7), exercise interventions (n = 5), cognitive behavioral therapy interventions (including cognitive therapy and behavioral therapy individually, n = 4), mindfulness-based interventions (n = 3), and virtual reality-based interventions (n = 2). In addition, each of the following interventions was reported in only one study: multidimensional physiotherapy, hypnosis, pain processing therapy, cognitive functional therapy, emotional awareness, fear-avoidance model treatment, electroacupuncture, spinal manipulative therapy, massage, and anodal transcranial direct current stimulation. Among the 20 studies, 15 assessed the association between pain catastrophizing and pain intensity, 11 focused on the association between pain catastrophizing and disability, 3 looked at the association between catastrophizing and pain interference, and 1 reported the association between pain catastrophizing and physical function.

### Quality Assessment

The results of the quality assessment using the Cochrane RoB 2 tool are shown in Table 2. Sixteen of the twenty studies were considered to raise some concern about the risk of bias in the overall assessment, and fifteen of them were due to insufficient information about measurements of the outcome and/or the selection of reported results. Concerns related to the measurements of the outcome were raised because outcome assessors (i.e., study participants) were aware of what intervention the participants received. Also, most of the concerns in the selection of reported results arose from the lack of information about how the reported results were selected.

### Associations Between Pain Catastrophizing and Pain-related Outcomes

#### Pain catastrophizing and pain intensity

Of the 20 studies, 15 studies evaluated the association between pain catastrophizing and pain intensity (Ashar et al., 2022; Bellomo et al., 2020; Bemani et al., 2023; Burns et al., 2024; Cashin et al., 2023; Čeko et al., 2024; Day et al., 2020; Gevers-Montoro et al., 2024; Gibbs et al., 2022; Kong et al., 2020; Matheve et al., 2020; Rizzo et al., 2021; Shaygan et al., 2022; Van Bogaert et al., 2023; Wood et al., 2023). The measures used to assess pain intensity were the Numeric Rating Scale (n = 11), Brief Pain Inventory (n = 2), Patient-Reported Outcomes Measurement Information System (PROMIS) Pain Intensity T-score (n = 1), and Visual Analog Scale (n = 1). Among these fifteen studies, seven conducted education-related interventions, four conducted exercise-based interventions, three applied cognitive behavioral therapy, two evaluated a virtual reality-based intervention (i.e., neuroscience-based therapy or exercise), two used a mindfulness-based intervention, and one study each applied multidimensional physiotherapy, hypnosis, pain processing therapy, emotional awareness, spinal manipulative therapy, or electroacupuncture intervention.

Regarding data analyses, seven studies employed linear regressions (Bellomo et al., 2020; Bemani et al., 2023; Gibbs et al., 2022; Kong et al., 2020; Matheve et al., 2020; Shaygan et al., 2022; Van Bogaert et al., 2023), and two of these seven studies showed a significant association between change in pain intensity and baseline PCS score (Bellomo et al., 2020; Shaygan et al., 2022), and one showed a significant association between baseline of pain intensity and pain catastrophizing (Van Bogaert et al., 2023). Four studies hypothesized that pain catastrophizing was a mediator of pain intensity (Ashar et al., 2022; Cashin et al., 2023; Rizzo et al., 2021; Wood et al., 2023), and three of the four studies reported that pain catastrophizing had a significant mediation role in pain intensity (Cashin et al., 2023; Rizzo et al., 2021; Wood et al., 2023). Two studies showed that pain catastrophizing mediated the effect of treatment on pain intensity (Cashin et al., 2023; Wood et al., 2023), and one study found that pain catastrophizing mediated the effect of treatment on pain intensity if the mediator and outcome were measured cross-sectionally (Rizzo et al., 2021). Moderation analyses were employed in five studies (Burns et al., 2024; Gevers-Montoro et al., 2024; Kong et al., 2020; Matheve et al., 2020), and two of these five reported that the baseline PCS score significantly moderated pain intensity (Burns et al., 2024; Kong et al., 2020). Finally, four studies (Ashar et al., 2022; Čeko et al., 2024; Day et al., 2020; Kong et al., 2020) evaluated the correlation between pain intensity and pain catastrophizing, but none reported significant correlations.

#### Pain catastrophizing and disability

Of the 20 studies, 11 studies assessed associations between pain catastrophizing and disability (Bohman et al., 2019; Cashin et al., 2023; Caumo et al., 2022; Chen et al., 2023; Gevers-Montoro et al., 2024; Gibbs et al., 2022; Hancock et al., 2024; Kong et al., 2020; Ryum & Stiles, 2023; Van Bogaert et al., 2023; Wood et al., 2023). The measures used to assess disability were the Roland-Morris Disability Questionnaire (n = 4) and a modified version (n = 1), Oswestry Disability Index (n = 2), normalized Neck Disability Index (n = 1), Work and Social Adjustment Scale (n = 1), Profile of Chronic Pain: Screen (n = 1), and Pain Disability Index (n = 1). For interventions, four studies evaluated an exercise intervention, three studies used cognitive or behavioral therapy interventions, three studies applied an education intervention, two studies used a mindfulness-based intervention, and one study each used an anodal transcranial direct current stimulation, spinal manipulative therapy, electroacupuncture, fear-avoidance model treatment, or massage intervention.

Among these eleven studies, five studies used linear regressions (Bohman et al., 2019; Caumo et al., 2022; Chen et al., 2023; Gibbs et al., 2022; Van Bogaert et al., 2023); two of the five studies showed a significant association between change in disability and change in pain catastrophizing (Caumo et al., 2022; Chen et al., 2023) and one showed a significant association between baseline of disability and pain catastrophizing (Van Bogaert et al., 2023). In addition, three studies included mediator analysis, and all three studies reported a significant mediation role of pain catastrophizing in disability (Cashin et al., 2023; Ryum & Stiles, 2023; Wood et al., 2023). Among the three studies using mediator analysis, two studies showed that pain catastrophizing mediated the effect of treatment on disability (Cashin et al., 2023; Wood et al., 2023), and the final study found that pain catastrophizing was a mediator between pain intensity and disability (Ryum & Stiles, 2023). Furthermore, four studies employed moderation analyses (Chen et al., 2023; Gevers-Montoro et al., 2024; Hancock et al., 2024; Kong et al., 2020), and one of these studies reported that change in pain catastrophizing significantly moderated change in disability (Gevers-Montoro et al., 2024). A final study included correlation analyses but showed no significant correlations (Kong et al., 2020).

#### Pain catastrophizing and pain interference

Of the twenty studies, three studies investigated the association between pain catastrophizing and pain interference (Burns et al., 2024; Čeko et al., 2024; Day et al., 2020). The measures used for assessing pain interference were the Multidimensional Pain Inventory-Pain Interference subscale (n = 1), Brief Pain Inventory Short Form Pain Interference subscale (n = 1), and PROMIS Pain Interference T-score (n = 1). Two of the studies used cognitive and/or behavioral therapy and mindfulness-based therapy interventions, and one study used a virtual reality neuroscience-based therapy intervention.

Among the three studies of pain interference, one study included a moderator analysis (Burns et al., 2024), two studies included correlation analyses (Čeko et al., 2024; Day et al., 2020), and one study included a mediator analysis (Čeko et al., 2024). All three studies showed a significant association between pain catastrophizing and pain interference. Results from two of the studies showed that changes in pain catastrophizing were significantly correlated with changes in pain interference (Čeko et al., 2024; Day et al., 2020). In addition, the study using mediator analysis showed that changes in pain catastrophizing significantly mediated the association between treatment (i.e., virtual reality neuroscience-based therapy) and change in pain interference (Čeko et al., 2024). Furthermore, the mediator analysis study showed a significant interaction between baseline PCS score and time point of observation for change in pain interference in the usual care group (Burns et al., 2024). However, the cognitive therapy, behavioral therapy, and mindfulness therapy groups in this study showed no significant interactions.

#### Pain catastrophizing and physical function

One study evaluated the association between PCS score and physical function, using the 36-item Short-Form Health Survey Physical Function subscale (n = 1) (Ryum & Stiles, 2023). This study used fear-avoidance model treatment, with and without exposure to feared movements, as an intervention. Change in PCS score was examined as a mediator between pain intensity and physical function; however, no significant association was observed.

### Limitations of the Included Studies

Table 1 describes the limitations as well as the future implications reported in each of the reviewed articles. Small sample sizes, homogeneity of participants, and lack of longer follow-up times were three major limitations across these studies. In general, the included research articles suggested that future studies should recruit a diverse and broad population to extend generalizability, include a larger sample size to secure the power to detect associations, measure more comprehensive aspects of indicators relevant to pain to find further associations between pain-related outcomes and other factors, and include longer follow-up of participants to investigate the chronological relationship between pain catastrophizing and pain over time.

## Discussion

This systematic review aimed to provide RCT-based information on observed associations between pain catastrophizing and four pain-related outcomes–pain intensity, disability, pain interference, and physical function–based on recent studies in adults with chronic musculoskeletal pain. Pain catastrophizing is a well-established predictor of the occurrence of chronic pain and related outcomes such as pain intensity and disability (Burns et al., 2015; Martinez-Calderon, Jensen, et al., 2019). Most of the studies included in this review examined either baseline pain catastrophizing or changes in pain catastrophizing as a predictor, mediator, or moderator of outcomes; most reported that both baseline and changes in pain catastrophizing were associated with changes in pain intensity, disability, and pain interference after intervention. Many of the findings of this review were consistent with those reported in the previous systematic review (Martinez-Calderon, Jensen, et al., 2019). Pain catastrophizing appears to be a modifiable factor through interventions and can be considered a precursor of and an interactive factor for pain-related outcomes, highlighting the importance of routinely assessing and addressing pain catastrophizing in adults with chronic musculoskeletal pain.

In this review of RCTs, three out of seven studies on pain intensity and three out of five studies on disability reported pain catastrophizing as a significant predictor, regardless of intervention type. In comparison, the previous review reported over 80% of longitudinal studies with significant associations between pain catastrophizing and pain intensity and disability (Martinez-Calderon, Jensen, et al., 2019). This discrepancy in the proportion of the studies showing significant associations may be attributed to differences between study designs (Kimachi et al., 2021; McDonald et al., 2023), controlling factors, and populations. Still, it also raises the possibility that the strength of these associations may be weaker than previously reported.

This review incorporated recent intervention studies and systematically examined the role of pain catastrophizing as a predictor, as well as a potential mediator and/or moderator of treatment effects. Notably, the majority of studies that assessed pain catastrophizing as a mediator reported it to be a significant mediator of treatment outcomes. In contrast, only a limited number of studies reported pain catastrophizing as a significant moderator. While studies employing behavioral therapy, cognitive therapy, and mindfulness-based stress reduction demonstrated significant moderating effects of baseline levels of pain catastrophizing on pain intensity and pain interference (Burns et al., 2024), other interventions, such as manipulation, exercise, and electroacupuncture, did not show a significant moderating role of pain catastrophizing on pain intensity (Gevers-Montoro et al., 2024; Kong et al., 2020; Matheve et al., 2020). In summary, the current review strengthens the evidence that pain catastrophizing primarily acts as a mediator rather than a moderator between treatment and pain-related outcomes among adults with chronic musculoskeletal pain, although it may partly serve as a moderator in psychological interventions. Findings regarding pain catastrophizing as a mediator versus moderator are consistent with findings from a previous review that focused on the association between pain catastrophizing and treatment outcomes. However, that review was limited to participants with nonspecific low back pain (Wertli et al., 2014). These results suggest that measuring pain catastrophizing before intervention may be effective to provide optimized interventions and capture the transition of the pain-related outcomes.

This distinction in the moderating role of pain catastrophizing across interventions may be attributable to differences in the therapeutic targets addressed by each intervention. Psychological interventions, such as cognitive-behavioral therapy and mindfulness-based approaches, primarily aim to modify maladaptive pain-related cognitions (Hush, 2020; Nicholas, 2022). Within the framework of the biopsychosocial model of pain, it is well-established that psychological processes can exert a significant influence on pain perception (Nicholas, 2022). Given that pain catastrophizing constitutes a core component of the psychological domain of pain, it is plausible that it would indicate a moderating effect in interventions targeting cognitive and emotional regulation. In contrast, interventions such as manipulation and exercise are primarily designed to produce changes at the biological or physical level. These interventions typically do not engage with cognitive mechanisms directly. Therefore, the absence of a significant moderating effect of pain catastrophizing in such interventions may reflect a mismatch between the therapeutic mechanism and pain catastrophizing. In summary, this review strengthens the evidence that pain catastrophizing primarily acts as a mediator rather than a moderator between treatment and pain-related outcomes among adults with chronic musculoskeletal pain, although it may partly serve as a moderator in psychological interventions.

Notably, although few of the included studies examined the association between pain interference and pain catastrophizing, all such studies showed a significant association. These results may indicate that pain catastrophizing plays a more substantial role in the mechanism of change in pain interference than in other pain-related outcomes. Given that pain catastrophizing reflects a maladaptive cognitive process characterized by exaggerated negative appraisals of pain, it is plausible that pain catastrophizing has a more direct influence on pain interference than on other outcomes. Pain interference lacks a consistent definition across the literature (Wilson, 2014). While it is sometimes regarded as an aspect of physical functioning (Dworkin et al., 2005), it is more commonly defined as the degree to which pain disrupts an individual’s physical, mental, and social activities (Amtmann et al., 2010). In contrast, the measurement of disability is specifically designed to evaluate physical impairments (Roland & Fairbank, 2000). Although the degree of disability correlates with quality of life (Mutubuki et al., 2020), disability and pain interference, while related, should be regarded as distinct concepts. The results of this review indicate the importance of differentiating these concepts.

Meanwhile, the association between physical function and pain catastrophizing was not found to be significant. Only one study of this relationship was eligible for inclusion; therefore, the current authors assume that there was an insufficient number of studies to infer the association. Previous observational research has shown associations between physical function and pain catastrophizing (Birch et al., 2019; Varallo et al., 2022); however, the evidence remains inconclusive. In addition, although all kinds of chronic musculoskeletal pain were included as eligible criteria, most studies focused on chronic back pain. This result is consistent with prior reviews (Martinez-Calderon, Jensen, et al., 2019; Rogers & Farris, 2022). This limited the generalizability of findings. Hence, future RCTs should consider including physical function measures to assess the association between physical function and pain catastrophizing, covering a broader range of different populations with chronic musculoskeletal pain, or focusing on musculoskeletal pain other than back pain, to obtain further generalizability.

### Strengths and Limitations

The strengths of this review are that only studies using data from RCTs were included and that associations between pain catastrophizing and pain-related outcomes were comprehensively examined. While observational studies cannot avoid potential confounders, RCTs can minimize the risk of confounders and clarify cause-and-effect relationships (Hariton & Locascio, 2018). Furthermore, this review synthesized findings across multiple previous systematic reviews, thereby strengthening the current body of evidence and providing up-to-date insights into the complex relationships between pain and pain catastrophizing.

However, this systematic review has several limitations as well. First, only research articles published in English were included, which may have excluded relevant studies in other languages. In addition, although the current authors referred to previous systematic reviews and were supported by an expert health science librarian to build search strategies across six databases, relevant articles or gray literature may still have been missed. Second, while the inclusion of only studies using RCT data ensures methodological rigor, several of the included studies were secondary analyses of RCTs. Therefore, the possibility that hypotheses were generated after the results were known cannot be excluded, which may introduce the risk of post-hoc bias. Finally, the studies included in this review covered a wide range of topics, including moderation, mediation, correlations, and associations, as well as multiple types of interventions. This heterogeneity made it difficult to synthesize the data using a meta-analysis.

## Conclusions

This systematic review provides a comprehensive overview of the association between pain catastrophizing and pain intensity, disability, pain interference, and physical function among adults with chronic musculoskeletal pain. The findings reported here strengthen the evidence that pain catastrophizing plays a crucial role in mediating treatment effects in chronic pain. Overall, the findings of this systematic review are consistent with previous reviews and extend the evidence of the association between pain catastrophizing and pain-related outcomes as a predictor, mediator, and moderator. Further evidence is required to identify what treatments might be most effective for patients with chronic musculoskeletal pain who experience pain catastrophizing.

## Supplementary Material

1

Supplementary material associated with this article can be found, in the online version, at doi:10.1016/j.pmn.2025.07.014.

## Figures and Tables

**Table 1:** Characteristics of Included Studies.

No	Author (Year)	Design	Aim	Population, Pain Type, and Sample Size	Intervention(s)/Control(s)	Measurement of Pain-related Outcome(s)	Association Between Pain Catastrophizing and Pain Outcomes	Limitations	Recommendations for Future Research
1	Ashar et al. (2022)	Parallel RCT	Examine the mechanisms of pain reprocessing therapy with mediation analyses	Adults (21–70 years) with chronic primary back pain (≥6 months) (N = 151)	Pain reprocessing therapy/ placebo/ usual care	Pain intensity: NRS (0–10)	**Mediation** DV: Change of outcome variable IV: Pain catastrophizing at follow-up Intensity: NA, N.S. **Correlation** Between changes of variables Intensity: NA, N.S.	Homogeneity of participant characteristics (well-educated, active, and long-standing low-to-moderate pain and disability) Limit to generalizability due to expert treatments	Test generalizability to other patient populations, therapists, and treatment contexts Test efficacy of intervention relative to leading psychological and medical treatments
2	Bellomo et al. (2020)	Secondary analysis of RCT	Examine whether baseline pain sensitivity predicts differential treatment responses	Females with fibromyalgia (N = 196)	Emotional awareness and expression therapy/ cognitive behavioral therapy/ fibromyalgia education	Pain intensity: BPI (0–10)	**Linear model** DV: Change of outcome variable IV: Pain catastrophizing at baseline **Intensity:** B = −0.21, positive	Homogeneity of participant characteristics (females, white, pain tolerance, and well-educated)	Conduct studies with more diverse and larger samples using multiple methods
3	Bemani et al. (2023)	Parallel RCT	Examine the superiority of multidimensional physiotherapy compared with usual physiotherapy among people with nonspecific chronic low back pain	Adults (18–50 years) with chronic nonspecific low back pain (N = 70)	Multidimensional physiotherapy (psychoeducation, graded exposure, postural correction, and electrotherapy)/traditional back school education	Pain intensity: NRS (0–10)	**Linear model** DV: Change of outcome variable IV: Pain catastrophizing at baseline **Intensity:** NA, N.S.	Homogeneity of participant characteristics (nonspecific chronic low back pain, moderate pain, moderate-to-severe disability, kinesiophobia) Medium-term follow-up Wide range of confidence intervals	Conduct long-term follow-up trial with a large sample size
4	Bohman et al. (2019)	Secondary analysis of RCT	Develop predictive models for short- and long-term clinically important improvement in females with chronic disabling neck pain in the clinical course of physiotherapy	Females (25–60 years) with chronic nonspecific disabling neck pain (≥3 months) (N = 86)	Neck coordination exercise/strength training/massage/usual care	Disability: Normalized NDI (0–100)	**Linear model** DV: Change of outcome variable IV: Pain catastrophizing at baseline **Disability:** *β* = 0.05, N.S.	Small sample size for a prediction study Unmeasured confounder due to secondary analysis Lots of attrition Possible selection bias	Examine external validity
5	Burns et al. (2024)	Parallel RCT	Identify treatment response predictors and moderators based on hypotheses from the Limit, Activate, and Enhance (LA&E) model	Adults (18–80 years) with chronic musculoskeletal pain of the low back or leg (≥6 months) (N = 521)	Behavioral therapy/cognitive therapy/mindfulness-based stress reduction/usual care	Pain intensity: NRS (0–10) Pain interference: MPI-PI (0–6)	**Moderation** DV: Change of outcome variable IV: Pain catastrophizing at baseline **Intensity:** NA, positive **Interference:** NA, positive	Possible reporting bias Unmeasured confounder	Use actigraphy measures and/or laboratory assessments of movement tasks
6	Cashin et al. (2023)	Secondary analysis of RCT	Examine mediation mechanisms in the effect of education and graded sensorimotor retraining intervention on pain and disability	Adults (18–70 years) with chronic low back pain (≥3 months) (N = 276)	Education and graded sensorimotor retraining/sham control	Pain intensity: NRS (0–10) Disability: RMDQ (0–24)	**Mediation** DV: Outcome variable 18 weeks after randomization IV: Pain catastrophizing at postintervention **Intensity:** B = −0.49, positive **Disability:** B = −1.06, positive	A certain amount of missing data Unmeasured confounder Unclearness of clinical meaningfulness	Collect mediator data at shorter intervals during treatment
7	Caumo et al. (2022)	Parallel RCT	Examine the effect of anodal transcranial direct current stimulation in reducing pain catastrophizing, disability, and other outcomes	Females (30–65 years) with fibromyalgia (N = 48)	Anodal transcranial direct current stimulation/sham-transcranial direct current stimulation	Disability: PCP:S (0–91)	**Linear model** DV: Change of outcome variable IV: Change of pain catastrophizing **Disability:** B = 0.16, positive (rumination); B = 0.39, positive (magnification)	Homogeneity of participant characteristics (females) Limit to generalizability due to lack of comparison with different stimulation sites and different parameters	Consider differential effects related to pain threshold
8	Čeko et al. (2024)	Parallel RCT	Assess the effectiveness of a novel virtual reality neuroscience-based therapy in reducing pain intensity and interference and improving functioning among individuals with chronic back pain and examine whether pain catastrophizing and kinesiophobia mediate the effects of intervention on pain outcomes	Adults (21–70 years) with chronic back pain (≥6 months) (N = 61)	Virtual reality neuroscience-based therapy/usual care	Pain intensity: BPI SF (0–10) Pain interference: BPI SF (0–10)	**Mediation** DV: Change of outcome variable IV: Change of pain catastrophizing **Interference:** B = −0.16, positive **Correlation** Between changes of variables **Intensity:** *r* = 0.15, N.S. **Interference:** *r* = 0.35, positive	Small sample size Homogeneity of participant characteristics (white) Lack of long-term follow-up	Conduct studies with more diverse samples, longer follow-ups, and diverse arms
9	Chen et al. (2023)	Secondary analysis of RCT	Identify what baseline demographic is associated with differential benefits from different interventions and examine other possible moderators and nonspecific predictors of treatment response by exploratory analyses	Adults (20–70 years) with chronic nonspecific low back pain (≥3 months) (N = 297)	Cognitive behavioral therapy/mindfulness-based stress reduction/usual care	Disability: mRMDQ (0–23)	**Linear model** DV: Change of outcome variable IV: Pain catastrophizing at baseline **Disability:** B = 0.12, positive **Moderation** DV: Change of outcome variable IV: Pain catastrophizing at baseline **Disability:** NA, N.S.	Limit to generalizability due to differences in exposure to intervention among the same group Limit to generalizability due to group intervention	Conduct further investigation considering the finding
10	Day et al. (2020)	Secondary analysis of pilot RCT	Explore potential mechanisms of intervention	Adults (≥18 years) with chronic low back pain (≥3 months) (N = 69)	Cognitive therapy/mindfulness meditation/mindfulness-based cognitive therapy	Pain intensity: NRS (0–10) Paininterference: T-score of PROMIS Pain Interference	**Correlation** Between variables at baseline **Intensity:** *r* = 0.01, N.S. **Interference:** *r* = −0.04, N.S. Between changes of variables **Intensity:** *r* = 0.21, N.S. **Interference:** *r* = 0.38, positive	Small sample size Limit of generalizability due to group-based analyses	Examine the relation between the treatment mechanisms and attention control or placebo effects Conduct long-term follow-up Perform moderator/mediator studies
11	Gevers-Montoro et al. (2024)	Parallel RCT	Assess processes underlying neoplastic pain and associated factors in patients with chronic primary low back pain treated with spinal manipulative therapy to determine how they contribute to chronic primary low back pain and its clinical improvement	Adults (18–70 years) with chronic primary low back pain (≥3 months) (N = 148)	Spinal manipulative therapy/ validated placebo spinal manipulative therapy/ healthy control	Pain intensity: NRS (0–100) Disability: ODI (0–50)	**Moderation** DV: Change of outcome variable IV: Change of pain catastrophizing **Intensity:** *β* = 0.16, N.S. **Disability:** *β* = 0.47, positive	Homogeneity of participant characteristics (low disability) Potential floor effect on disability Potential performance bias	Confirm the mechanism of improvement of chronic primary low back pain by spinal manipulative therapy
12	Gibbs et al. (2022)	Parallel RCT	Compare the effect of a general trunk-focused calisthenic exercise intervention with a powerlifting-style resistance training model, both paired with consistent pain education, for people with chronic low back pain	Adults (18–65 years) with chronic primary low back pain (N = 64)	Powerlifting + pain education/body weight exercise + pain education	Pain intensity: VAS (100 mm) Disability: ODI (0–50)	**Linear model** DV: Outcome variable at time points IV: Pain catastrophizing at postintervention Week 8 **Intensity:** B = 0.03, N.S. **Disability:** B = 0.21, N.S. Week 12 **Intensity:** B = 0.05, N.S. **Disability:** B = 0.07, N.S. Week 24 **Intensity:** B = 0.06, N.S. **Disability:** B = 0.14, N.S.	Potential selection bias Small sample sizes for causal analysis Lack of individualization of treatments	Conduct a study with a larger sample for exploratory regression analysis Explore the effectiveness of individualization through multiarm interventions
13	Hancock et al. (2024)	Secondary analysis of RCT	Identify factors that respond best to CFT compared with usual care	Adults (≥18 years) with chronic low back pain (≥3 months) (N = 492)	Cognitive functional therapy/usual care	Disability: RMDQ (0–24)	**Moderation** DV: Change of outcome variable IV: Change of pain catastrophizing Week 13 **Disability:** *β* = −0.02, N.S. Week 52 **Disability:** *β* = −0.04, N.S.	N/A	Investigate with larger samples for moderator analysis
14	Kong et al. (2020)	Parallel RCT	Investigate the effectiveness of electroacupuncture in patients with chronic low back pain and identify factors associated with treatment response	Adults (18–65 years) with chronic low back pain (≥6 months) (N = 121)	Electroacupuncture/sham-electro stimulate	Pain intensity: T-score of PROMIS Pain Intensity Disability: RMDQ (0–24)	**Linear model** DV: Outcome variable at time point IV: Pain catastrophizing at baseline **Intensity:** *β* = −0.16, N.S. **Moderation** DV: Outcome variable at the time point IV: Pain catastrophizing at baseline **Intensity:** *β* = 0.26, N.S. **Disability:** *β* = −0.11, N.S. **Correlation** Between changes of outcome variables and pain catastrophizing at baseline **Intensity:** *r* = 0.23, N.S. **Disability:** *r* = −0.08, N.S.	Lack of quantification of the specific effect of intervention Missing data on masking due to researchers’ mistake Lack of follow-up	Collect longer follow-up data
15	Matheve et al. (2020)	Parallel RCT	Investigate the effectiveness of VR distraction on pain intensity and the influence of baseline pain intensity, pain-related fear, and pain catastrophizing on its effectiveness	Adults (18–65 years) with chronic nonspecific low back pain (≥3 months) (N = 82)	Virtual reality pelvic tilt exercises/pelvic tilt exercises	Pain intensity: NRS (0–10)	**Linear model** DV: Outcome variable at time point IV: Pain catastrophizing at baseline **Intensity:** B = −0.05, positive **Moderation** DV: Outcome variable at the time point IV: Pain catastrophizing at baseline (dichotomized to high/low) **Intensity:** NA, N.S. DV: Outcome variable at the time point IV: Pain catastrophizing at baseline (dichotomized to high/low) **Intensity:** *β* = −0.002, N.S.	Lack of follow-up Homogeneity of participant characteristics (mild-to-moderate baseline pain intensity, familiar with pelvic tilts) Small sample size for moderator analyses Lack of adjustment in the analysis Lack of masking (participants and researchers)	Investigate with longer follow-up Apply direct measures of attention to pain or experimental methods Conduct a study with a larger sample
16	Rizzo et al. (2021)	Secondary analysis of RCT	Clarify the causal role of pain catastrophizing on pain intensity by mediation analysis	Adults (18–80 years) with chronic nonspecific low back pain (≥3 months) (N = 100)	Pain education using the book *Explain Pain* + hypnosis enhancement/pain education using the book *Explain Pain*	Pain intensity: NRS (0–10)	**Mediation** DV: Outcome variable at follow-up IV: Pain catastrophizing at postintervention **Intensity:** B = −0.10, N.S. DV: Outcome variable at follow-up IV: Pain catastrophizing at follow-up **Intensity:** B = −0.62, positive	Potential confounders due to post-hoc analysis	Conduct longitudinal assessments for mediator analysis on pain and disability
17	Ryum and Stiles (2023)	Secondary analysis of pilot RCT	Examine effect of an exposure-based treatment for chronic low back pain and role of pain catastrophizing, fear-avoidance beliefs, and pain self-efficacy as mediators of changes in pain intensity and disability	Adults (18–60 years) with chronic low back pain (≥3 months) (N = 69)	Fear-avoidance model treatment with in-session exposure/fear-avoidance model treatment without in-session exposure	Disability: WSAS (0–40) Physical function: SF-36 PF (0–100)	**Mediation** DV: Change of outcome variable IV: Change of pain catastrophizing **Disability:** *β =* 0.37, positive **Physical function:** *β* = −0.02, N.S.	Unmeasured confounders due to secondary analysis Small sample size for mediation analysis Limit to causal relationship interpretation due to cross-sectional analysis	Investigate relationship between pain self-efficacy and performance test Examine multiple mediators of treatment outcome simultaneously in clinical trials of chronic low back pain Apply findings to other chronic pain
18	Shaygan et al. (2022)	Parallel RCT	Compare effects of multimedia and face-to-face pain management education on pain intensity and pain catastrophizing among participants with chronic low back pain	Adults (≥18 years) with chronic nonspecific low back pain (N = 90)	Face-to-face pain management education/multimedia pain management education/usual care	Pain intensity: NRS (0–10)	**Mediation** DV: Change of outcome variable IV: Change of pain catastrophizing **Intensity:** B = 0.068, positive	Unmeasured outcomes (disability and physical function) Potential reporting bias Lack of follow-up Limit to compatibility due to lack of cutoff in pain catastrophizing Small sample size	Conduct a study with a larger sample Investigate with longer follow-up Collect broader aspects of pain, such as functionality, sleep, and mobility, using the BPI and the West Haven-Yale Multidimensional Pain Inventory
19	Van Bogaert et al. (2023)	Secondary analysis of RCT	Explore and validate a path model using an SEM analysis to explain variables’ contributions to health-related quality of life and functional status among people with chronic nonspecific spinal pain	Adults (18–65 years) with chronic nonspecific spinal pain (neck pain or back pain) (≥3 months) (N = 120)	Modern neuroscience education/traditional back and neck school education and general exercises	Pain intensity: NRS (0–10) Disability: PDI (0–70)	**Linear model** DV: Outcome variables at baseline IV: Pain catastrophizing at baseline **Intensity:** *β* = 0.27, positive **Disability:** *β* = 0.17, positive	Unmeasured confounder due to secondary analysis Small sample size for SEM analysis Possible selection bias Limit to causal relationship interpretation due to cross-sectional analysis	Include more predictors in path model with a larger sample study
20	Wood et al. (2023)	Secondary analysis of RCT	Determine whether changes in pain catastrophizing and kinesiophobia mediate effect of Pilates exercise on pain intensity and physical function by causal mediation analysis	Adults (18–80 years) with chronic nonspecific low back pain (≥3 months) (N = 255)	Pilates (1×/week)/Pilates (2×/week)/Pilates (3×/week)/providing booklet about low back pain	Pain intensity: NRS (0–10) Disability: RMDQ (0–24)	**Mediation** DV: Outcome variable at postintervention IV: Pain catastrophizing at postintervention **Intensity:** B = −0.21, positive **Disability:** B = −0.64, positive	Undesigned post-hoc mediation analysis Decreased sample size due to missing data Unmeasured confounder	Conduct fully powered RCT

Positive indicates there is a significant association between pain catastrophizing and pain-related outcomes. B represents the unstandardized beta coefficient, whereas *β* represents the standardized beta coefficient. In mediation analysis, the beta coefficient shows the indirect effect of the mediator.

Abbreviations: BPI = Brief Pain Inventory; BPI SF = Brief Pain Inventory Short Form; DV = dependent variable; IV = independent variable; MPI-PI = Multidimensional Pain Inventory-Pain Interference; mRMDQ = Modified Roland-Morris Disability Questionnaire; NA = not available; NDI = Neck Disability Index; NRS = numerical rating scale; N.S. = no significant association between pain catastrophizing and pain-related outcomes; ODI = Oswestry Disability Index; PCP:S = Profile of Chronic Pain: Screen; PDI = Pain Disability Index; PROMIS = Patient-Reported Outcomes Measurement Information System; RCT = randomized controlled trial; RMDQ = Roland-Morris Disability Questionnaire; SEM = structural equation modeling; SF-36 PF = 36-Item Short Form Health Survey Physical Function; VAS = Visual Analog Scale; WSAS = Work and Social Adjustment Scale.

**Table 2:** Risk-of-bias Assessments of the Included Studies

Author (Year)	Randomization Process	Deviations from Intended Interventions	Missing Outcome Data	Measurement of Outcome	Selection of Reported Result	Overall
Ashar et al. (2022)	Some concerns	Low risk	Low risk	Some concerns	Low risk	Some concerns
Bellomo et al. (2020)	Low risk	Low risk	Low risk	Some concerns	Some concerns	Some concerns
Bemani et al. (2023)	Low risk	Low risk	Low risk	Low risk	Low risk	Low risk
Bohman et al. (2019)	Some concerns	Some concerns	Low risk	Some concerns	Low risk	Some concerns
Burns et al. (2024)	Low risk	Low risk	Some concerns	Some concerns	Low risk	Some concerns
Cashin et al. (2023)	Low risk	Some concerns	Low risk	Low risk	Low risk	Some concerns
Caumo et al. (2022)	Low risk	Low risk	Low risk	Low risk	Some concerns	Some concerns
Čeko et al. (2024)	Low risk	Low risk	Low risk	Some concerns	Low risk	Some concerns
Chen et al. (2023)	Low risk	Some concerns	Low risk	Some concerns	Low risk	Some concerns
Day et al. (2020)	Low risk	Low risk	Some concerns	Some concerns	Some concerns	Some concerns
Gevers-Montoro et al. (2024)	Low risk	Low risk	Low risk	Low risk	Low risk	Low risk
Gibbs et al. (2022)	Low risk	Low risk	Low risk	Low risk	Some concerns	Some concerns
Hancock et al. (2024)	Low risk	Low risk	Low risk	Some concerns	Low risk	Some concerns
Kong et al. (2020)	Low risk	Low risk	Low risk	Low risk	Low risk	Low risk
Matheve et al. (2020)	Low risk	Low risk	Some concerns	Some concerns	Some concerns	Some concerns
Rizzo et al. (2021)	Low risk	Low risk	Low risk	Some concerns	Some concerns	Some concerns
Ryum and Stiles (2023)	Low risk	Low risk	Some concerns	Some concerns	Some concerns	Some concerns
Shaygan et al. (2022)	Low risk	Low risk	Low risk	Low risk	Low risk	Low risk
Van Bogaert et al. (2023)	Low risk	Low risk	Low risk	Low risk	Some concerns	Some concerns
Wood et al. (2023)	Low risk	Low risk	Low risk	Some concerns	Some concerns	Some concerns

## Key Practice Points

- Pain catastrophizing is considered a critical psychological factor associated with pain-related outcomes.
- This systematic review focused on randomized controlled trials to examine the current evidence of the association between pain catastrophizing and pain-related outcomes (e.g., pain intensity, disability, pain interference, and physical function) among adults with chronic musculoskeletal pain.
- The results showed that pain catastrophizing plays a crucial role in mediating treatment effects on pain-related outcomes in chronic musculoskeletal pain.
