# Supplementary material for: A Systematic Review of Pain Catastrophizing and Chronic Musculoskeletal Pain
Source: Pain Manag Nurs. Author manuscript; Available in PMC 2026 Aug 7. (PMC13447084; doi:10.1016/j.pmn.2025.07.014)
Supplement: Supplementary file 1 [file NIHMS2191943-supplement-1.docx]

Supplemental file 1

**Embase – 2004 articles (Data extracted on 2024/10/1)**

('pain catastrophizing scale'/exp OR 'catastrophizing'/exp OR catastroph*:ab,ti,kw OR helplessness*:ab,ti,kw OR magnificat*:ab,ti,kw OR ruminat*:ab,ti,kw OR 'pain catastrophizing scale' OR 'coping strategies questionnaire')

AND

('fibromyalgia':ab,ti,kw OR 'fatigue syndrome':ab,ti,kw OR 'osteoarthritis':ab,ti,kw OR 'epicondylalgia':ab,ti,kw OR 'rheumatoid arthritis':ab,ti,kw OR 'osteoarthrosis':ab,ti,kw OR 'arthritis':ab,ti,kw OR 'spondyloarthritis':ab,ti,kw OR 'ankylosing spondylitis':ab,ti,kw OR 'temporomandibular':ab,ti,kw OR 'whiplash':ab,ti,kw OR 'rotator cuff':ab,ti,kw OR 'musculoskeletal disease*':ab,ti,kw OR 'musculoskeletal disorder*':ab,ti,kw OR 'shoulder pain':ab,ti,kw OR 'neck pain':ab,ti,kw OR 'back pain':ab,ti,kw OR 'elbow pain':ab,ti,kw OR 'hand pain':ab,ti,kw OR 'pelvic pain':ab,ti,kw OR 'orofacial pain':ab,ti,kw OR 'knee pain':ab,ti,kw OR 'hip pain':ab,ti,kw OR 'ankle pain':ab,ti,kw OR 'musculoskeletal pain':ab,ti,kw OR 'widespread pain':ab,ti,kw)

AND

('physical function'/exp OR 'physical function':ab,ti,kw OR 'physical performance'/exp OR 'physical performance*':ab,ti,kw OR 'disability'/exp OR disabilit*:ab,ti,kw OR 'pain interference'/exp OR interfer*:ab,ti,kw OR 'pain intensity'/exp OR 'pain severity'/exp OR sever*:ab,ti,kw OR intens*:ab,ti,kw)

AND [english]/lim

AND [01-10-2018]/sd NOT [02-10-2024]/sd

**Pubmed – 1171 articles (Data extracted on 2024/10/1)**

(catastroph*[Title/Abstract] OR helplessness*[Title/Abstract] OR magnificat*[Title/Abstract] OR ruminat*[Title/Abstract] OR “pain catastrophizing scale” OR “coping strategies questionnaire” OR "catastrophization"[MeSH Terms])

AND

((“fibromyalgia”[Title/Abstract] OR “fatigue syndrome”[Title/Abstract] OR “osteoarthritis”[Title/Abstract] OR “epicondylalgia”[Title/Abstract] OR “rheumatoid arthritis”[Title/Abstract] OR “osteoarthrosis”[Title/Abstract] OR “arthritis”[Title/Abstract] OR “spondyloarthritis”[Title/Abstract] OR “ankylosing spondylitis”[Title/Abstract] OR “temporomandibular”[Title/Abstract] OR “whiplash”[Title/Abstract] OR “rotator cuff” [Title/Abstract]) OR ("musculoskeletal disease"[Title/Abstract] OR "musculoskeletal diseases"[Title/Abstract] OR "musculoskeletal disorder"[Title/Abstract] OR "musculoskeletal disorders"[Title/Abstract]) OR ("shoulder pain"[Title/Abstract] OR "neck pain"[Title/Abstract] OR "back pain"[Title/Abstract] OR "elbow pain"[Title/Abstract] OR "hand pain"[Title/Abstract] OR "pelvic pain"[Title/Abstract] OR "orofacial pain"[Title/Abstract] OR "knee pain"[Title/Abstract] OR "hip pain"[Title/Abstract] OR "ankle pain"[Title/Abstract] OR "musculoskeletal pain"[Title/Abstract] OR "widespread pain"[Title/Abstract]))

AND

"physical performance*"[Title/Abstract] OR "physical function"[Title/Abstract] OR disabilit*[Title/Abstract] OR interfer*[Title/Abstract] OR sever*[Title/Abstract] OR intens*[Title/Abstract] OR "physical functional performance"[MeSH Terms] OR "pain measurement"[MeSH Terms]

AND

((2018/10/1:2024/10/1[pdat]) AND (english[Filter]))

**CINAHL – 479 articles (Data extracted on 2024/10/1)**

((MM "Catastrophization") OR (“pain catastrophizing scale“ OR “coping strategies questionnaire“) OR TI (catastroph* OR helplessness* OR magnificat* OR ruminat*) OR AB (catastroph* OR helplessness* OR magnificat* OR ruminat*))

AND

((MM "Musculoskeletal Pain") TI("shoulder pain" OR "neck pain" OR "back pain" OR "elbow pain" OR "hand pain" OR "pelvic pain" OR "orofacial pain" OR "knee pain" OR "hip pain" OR "ankle pain" OR "musculoskeletal pain" OR "widespread pain") OR AB("shoulder pain" OR "neck pain" OR "back pain" OR "elbow pain" OR "hand pain" OR "pelvic pain" OR "orofacial pain" OR "knee pain" OR "hip pain" OR "ankle pain" OR "musculoskeletal pain" OR "widespread pain") OR TI(“musculoskeletal disease*” OR “musculoskeletal disorder*”) OR AB(“musculoskeletal disease*” OR “musculoskeletal disorder*”) OR TI(“fibromyalgia” OR “fatigue syndrome” OR “osteoarthritis” OR “epicondylalgia” OR “rheumatoid arthritis” OR “osteoarthrosis” OR “arthritis” OR “spondyloarthritis” OR “ankylosing spondylitis” OR “temporomandibular” OR “whiplash” OR “rotator cuff” ) OR AB(“fibromyalgia” OR “fatigue syndrome” OR “osteoarthritis” OR “epicondylalgia” OR “rheumatoid arthritis” OR “osteoarthrosis” OR “arthritis” OR “spondyloarthritis” OR “ankylosing spondylitis” OR “temporomandibular” OR “whiplash” OR “rotator cuff”))

AND

(TI("physical performance*" OR "physical function" OR disabilit* OR interfer* OR sever* OR intens*) OR AB("physical performance*" OR "physical function" OR disabilit* OR interfer* OR sever* OR intens*))

Limiters - Publication Date: 20181001-20241031; Language: English

**PsycoInfo – 1128 articles (Data extracted on 2024/10/1)**

(subject(Catastrophizing) OR tiab(catastroph* OR helplessness* OR magnificat* OR ruminat*) OR ("pain catastrophizing scale" OR "coping strategies questionnaire"))

AND

(tiab("shoulder pain" OR "neck pain" OR "back pain" OR "elbow pain" OR "hand pain" OR "pelvic pain" OR "orofacial pain" OR "knee pain" OR "hip pain" OR "ankle pain" OR "musculoskeletal pain" OR "widespread pain") OR tiab(“musculoskeletal disease*” OR “musculoskeletal disorder*”) OR tiab("fibromyalgia" OR "fatigue syndrome" OR "osteoarthritis" OR "epicondylalgia" OR "rheumatoid arthritis" OR "osteoarthrosis" OR "arthritis" OR "spondyloarthritis" OR "ankylosing spondylitis" OR "temporomandibular" OR "whiplash" OR "rotator cuff"))

AND

(tiab("physical performance*" OR "physical function" OR disabilit* OR interfer* OR sever* OR intens*) OR MAINSUBJECT.EXACT("Pain Perception") OR MAINSUBJECT.EXACT("Pain Measurement"))

AND

(la.exact("ENG") AND pd(20181001-20241001))

**Web of Science – 1095 articles (Data extracted on 2024/10/1)**

TS=(Catastrophizing) OR AB=(catastroph* OR helplessness* OR magnificat* OR ruminat*) OR TI=(catastroph* OR helplessness* OR magnificat* OR ruminat*) OR ALL=("pain catastrophizing scale" OR "coping strategies questionnaire")

AND

(TI=("fibromyalgia" OR "fatigue syndrome" OR "osteoarthritis" OR "epicondylalgia" OR "rheumatoid arthritis" OR "osteoarthrosis" OR "arthritis" OR "spondyloarthritis" OR "ankylosing spondylitis" OR "temporomandibular" OR "whiplash" OR "rotator cuff") OR AB=("fibromyalgia" OR "fatigue syndrome" OR "osteoarthritis" OR "epicondylalgia" OR "rheumatoid arthritis" OR "osteoarthrosis" OR "arthritis" OR "spondyloarthritis" OR "ankylosing spondylitis" OR "temporomandibular" OR "whiplash" OR "rotator cuff") OR TI=(“musculoskeletal disease*” OR “musculoskeletal disorder*”) OR AB=(“musculoskeletal disease*” OR “musculoskeletal disorder*”) OR TI=("shoulder pain" OR "neck pain" OR "back pain" OR "elbow pain" OR "hand pain" OR "pelvic pain" OR "orofacial pain" OR "knee pain" OR "hip pain" OR "ankle pain" OR "musculoskeletal pain" OR "widespread pain") OR AB=("shoulder pain" OR "neck pain" OR "back pain" OR "elbow pain" OR "hand pain" OR "pelvic pain" OR "orofacial pain" OR "knee pain" OR "hip pain" OR "ankle pain" OR "musculoskeletal pain" OR "widespread pain"))

AND

(TI=("physical performance*" OR "physical function" OR disabilit* OR interfer* OR sever* OR intens*) OR AB=("physical performance*" OR "physical function" OR disabilit* OR interfer* OR sever* OR intens*))

AND LA=(English) Timespan: 2018-10-01 to 2024-10-01

**Scopus – 1971 articles (Data extracted on 2024/10/2)**

( TITLE-ABS-KEY ( catastroph* OR helplessness* OR magnificat* OR ruminat* ) OR ("pain catastrophizing scale" OR "coping strategies questionnaire" ))

AND

(  TITLE-ABS-KEY ( “musculoskeletal disease*” OR “musculoskeletal disorder*” OR "shoulder pain" OR "neck pain" OR "back pain" OR "elbow pain" OR "hand pain" OR "pelvic pain" OR "orofacial pain" OR "knee pain" OR "hip pain" OR "ankle pain" OR "musculoskeletal pain" OR "widespread pain" OR "shoulder pain" OR "neck pain" OR "back pain" OR "elbow pain" OR "hand pain" OR "pelvic pain" OR "orofacial pain" OR "knee pain" OR "hip pain" OR "ankle pain" OR "musculoskeletal pain" OR "widespread pain" OR "fibromyalgia" OR "fatigue syndrome" OR "osteoarthritis" OR "epicondylalgia" OR "rheumatoid arthritis" OR "osteoarthrosis" OR "arthritis" OR "spondyloarthritis" OR "ankylosing spondylitis" OR "temporomandibular" OR "whiplash" OR "rotator cuff" ) )

AND

 ( TITLE-ABS-KEY ( "physical performance*" OR "physical function" OR disabilit* OR interfer* OR sever* OR intens*))

AND (PUBDATETXT ( "October 2018" OR "November 2018" OR "December 2018" ) OR ( PUBYEAR > 2019 ) )

AND ( LIMIT-TO ( LANGUAGE , "English" ) )
